# Risk of General Anesthesia in Pediatric Skin Procedures with Projection on Tumescent Anesthesia

**DOI:** 10.1155/2020/9327152

**Published:** 2020-05-30

**Authors:** Abdulmajeed Alajlan, Bader M. Alhabeeb, Ali M. Alhazmi, Osama A. Alobaid, Ahmed A. Alharthi, Nada I. Al-Habib, Ahmed M. El-Malky

**Affiliations:** ^1^Dermatology Department, Faculty of Medicine, King Saud University, Riyadh, Saudi Arabia; ^2^Resident Dermatology Department, King Salman Hospital, Riyadh, Saudi Arabia; ^3^Saudi Board of Anesthesia, Anesthesia Department, Security Force Hospital (SFH), Riyadh, Saudi Arabia; ^4^Quality Management Department, King Saud University Medical City, Riyadh, Saudi Arabia; ^5^Research Chair of Evidence-Based Healthcare and Knowledge Translation, Deputy Supervisor Morbidity and Mortality Review Unit, College of Medicine, King Saud University Medical City, Riyadh, Saudi Arabia; ^6^Public Health and Community Medicine Department, Theodor Bilharz Research Institute, Academy of Scientific Research, Cairo, Egypt

## Abstract

**Background:**

Uses of general anaesthesia in outpatient invasive procedures have increased, especially in dermatology. Being uncooperative, children often require general anaesthesia, since surgical skin operations are mostly painful.

**Aim:**

The purpose of this study is to evaluate the safety, significant adverse events, and the complication rates related to general anaesthesia, when used among pediatric population undergoing skin procedures.

**Methods:**

We conducted a first retrospective cohort study of patient chart review during the period from September 1, 2017 through September 2019. All patients admitted for pediatric skin procedures during this period have participated in our study. We reviewed selected charts to document any unexpected admissions, adverse events, or complications. Surgical outcomes and anaesthesia complications were reviewed by three anesthesiologists. We assessed inter-rater reliability.

**Results:**

A total of 211 procedures were reported for 211 patients with 19 diagnoses. No adverse events related to anaesthesia were recognized, apart from minor complications noticed in twelve patients. The kappa value range is between 0.78 and 1.00 (95% C.I., 0.46809 to 1.00).

**Conclusion:**

Dermatologist and pediatricians can safely do necessary procedures under general anaesthesia with the supervision of pediatric-trained anesthesiologists while considering other safety and risk precautions and the pediatric age group.

## 1. Introduction

Surgical skin procedures are usually performed as an outpatient service and accompanied by a few trivial complications [1]. A multicenter retrospective review found no serious complications among pediatric patients [2]. Recently, the use of general anaesthesia in pediatric skin procedures has increased [2].

It is challenging to mollify a conscious child during an invasive procedure, even with local anaesthesia. Surgeons often have to operate young children using general anaesthesia to minimize pain and psychological trauma associated with local anaesthesia and also to improve the overall outcome [2]. Randomized control trials evaluating general anaesthesia in pediatric invasive skin procedures in comparison with local anaesthesia or any other approach are sparse. We conducted this retrospective cohort study to highlight the safety of general anaesthesia in the pediatric population undergoing dermatologic procedures [3].

## 2. Materials and Methods

Patients were identified through electronic medical records of a tertiary-care hospital. Patients who had surgeries under general anaesthesia performed by a dermatologist during the period from September 1, 2017 through September 2019 were included in the study. An adverse event was defined as any complication related to the general anaesthesia during the operation or immediately postoperative or an unplanned readmission within one week of the operation due to a cause related to anaesthesia, either to the clinic or pediatric emergency department. Substantial adverse events were described as any complication which led to undesirable outcomes or medical intervention leading to extended length of stay. We conducted a retrospective file review to gather patients' demographic criteria such as age, gender, diagnosis and performed procedures, and unexpected admissions, incidents, or complications. We obtained the approval of the Institutional Review Board.

All the patients had an American Society of Anesthesiologists (ASA) status I or II. A pediatric anesthesiologist administered the general anaesthesia to all patients. All patients were provided strict instructions prohibiting them to eat or drink anything, two hours before the procedure, unless being an infant <5 months of age, in which case, only clear fluids were allowed. Easily digestible solids are not allowed 4 to 6 hours before surgery. Milk products were not allowed 8 hours before surgery. We used nitrous oxide, oxygen, and sevofluorane for anaesthesia induction and maintenance. We frequently administered other agents during the procedure, including ondansetron, ketorolac, and dolasetron. Morphine or fentanyl was also given. Midazolam and acetaminophen, solo or in combination with codeine, were also administered occasionally as premedication. Marcaine 0.25% was locally infiltrated around the surgical incision in case of excision-repair procedures. We gave our patients morphine or fentanyl postoperatively for pain control, if required. Ibuprofen or acetaminophen was prescribed as home medication upon discharge for pain control if necessary.

Postoperative complication severity was classified as minor or major and were assessed by three pediatric anaesthesia consultants to reach a consensus about the level of severity. Each anesthesiologist evaluated the postoperative complication against prespecified criteria [4] and gave a score from one to five (Likert scale). “One” was interpreted as strongly disagree and “five” as strongly agree.

We conducted inter-rater reliability tests (IRR) to determine the level of agreement between raters. We also used the intraclass correlation and measured the consistency of ratings because we have more than two raters. The inter-rater agreement was appraised by calculating the “Fleiss' kappa statistic” for each contributor [5]. The estimation of the statistic was established to be 1.00, when there was comprehensive agreement and “zero” when the rate was alike to that perceived by chance Table 1. The data revealed from the Likert scale were used in assessment of the level of agreement among raters by inter-rater reliability tests and determination of intraclass correlation coefficient (kappa value). We determined the level of percent agreement between raters.

The correlation between contributors' responses was statistically analyzed with the “chi-square nonparametric test,” with a level of significance of 5%, with 95% confidence interval (CI). The analyses were all prespecified, including the calculation of the minimum “*n*” and the “factor beta” statistical error, which was lower than 20%. The sample size is in congruence to the results and conclusion.

We performed statistical analyses with IBM-SPSS “version 20” software (IBM-Corp, Armonk, NY, USA). Descriptive data were described. Based on kappa correlation coefficient, the results were categorized as “excellent, very good, good, moderate, fair, and poor.”

## 3. Results

A total of 211 procedures under general anaesthesia were performed on 211 unique pediatric patients with a total of 19 dermatological diagnoses. The patients' age ranged from four months to 17 years of age, with a mean age of 4.7 years. A total of 111 (52.6%) patients were males, and 100 (47.3%) were females (Table 2).

Of the 211 procedures performed under general anaesthesia, 88 (41.6%) were flash lamp-pulsed dye laser and 123 (58.3%) were excisional surgical procedures. The most common diagnoses were congenital melanocytic nevi (23%), nevus sebaceous (21%), port wine stain (19%), infantile hemangioma (8%), dermoid cysts (7%), and pilomatricomas (4%). Other diagnoses (18%) are represented in Table 3.

A total of 12 of the 211 patients (5.6%) had clinically relevant complications related to anaesthesia (Table 4), of which four patients developed postoperative sore throat, nausea, and vomiting. Three patients developed mild-to-moderate postoperative frontal headache. Two patients developed postoperative minor trauma to the teeth and lips. One patient developed postoperative “Bruising” from IV injection. One patient developed postoperative painful neck muscles.

One patient experienced recall of unpleasant dreams and return to consciousness before completion of pharyngeal suction and extubating. No vascular complications such as thrombosis or thrombophlebitis were noted. No nerve complications arose from malpositioning of the patient on the operating table. No eye complications such as corneal abrasions were identified. There was no immediate intra- or postoperative complications such as bradycardia, tachycardia, and apnea in our patient population. No admissions for dehydration from nausea and vomiting were recorded.

**Figure 1:**
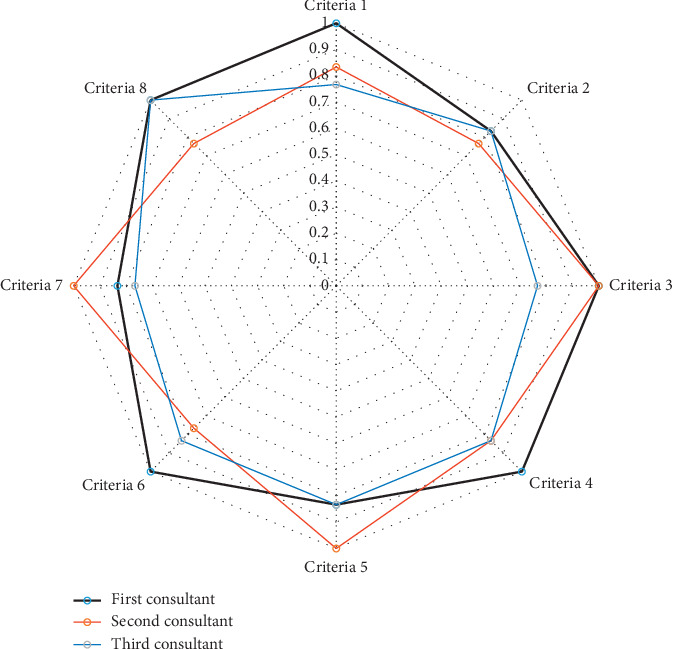
Percent agreement among raters for the eight prespecified criteria used for evaluation of the twelve patients.

**Figure 2:**
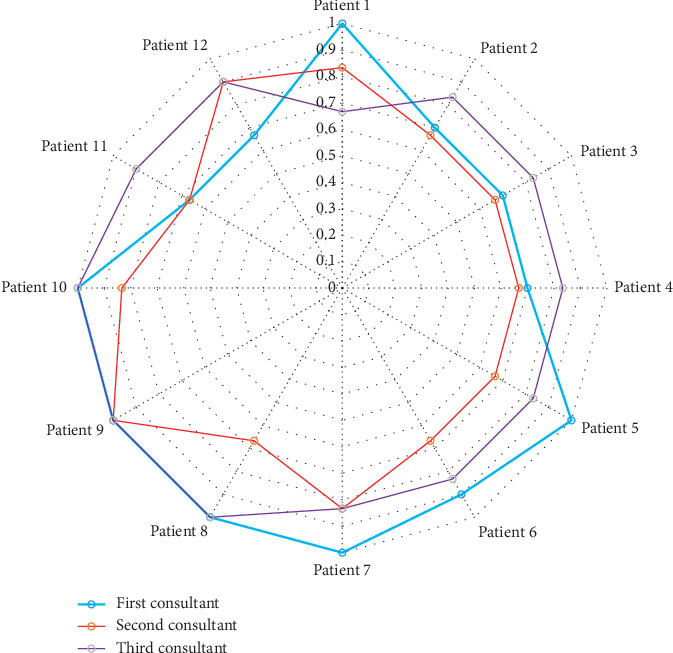
Percent agreement among raters for the twelve patients evaluated by the consultants in terms of complication criteria.

Three pediatric anesthesiologists evaluated the twelve patients with complications (Table 5). All the twelve patients had minor complications related to anaesthesia. Inter-rater reliability was high for every question in the evaluation criteria and for every patient. Most of the kappa values were between 0.70 and 1.00 (95% C.I., 0.46809 to 1.00) denoting very good-to-excellent agreement (Figures 1 and 2).

## 4. Discussion

The safety of paediatric general anaesthesia has been addressed up to the present [6]. However, recent epidemiological research studies concerning general anaesthesia-related complications particularly in pediatric skin surgeries were scarce [7]. The available literature is mostly on general anaesthesia in major operations such as neurosurgery, cardiovascular surgery, and abdominal surgery in adults and children [8]. Little number of articles have addressed the safety of general anaesthesia in pediatric dermatology. Morbidity and mortality from general anaesthesia in skin surgeries among children are very rare and clinically insignificant [8, 9].

The incidence of postoperative headache as a minor complication related to anaesthesia ranges between 2 and 70% [10]. Substantial reasons for this wide range are not apparent [11]. However, short-lived or mild headache may go unreported by the patient or observer [12]. Our results showed low incidence of headache in spite of the patient being interviewed. However, no serious complications or mortalities were noted.

The findings of our study are not similar but consistent with the foregoing publications. The complications of general anaesthesia after elective pediatric dermatologic operations were very low, as shown in a multicenter study that was conducted to evaluate the safety and adverse events of general anaesthesia which included 270 children between two months and eighteen years of age [2].

Another study found that laryngospasm and transient apnea were the most common complications noted; however, the results were statistically insignificant [13]. Our findings did not show any incidents of laryngospasm or apnea. Wound infection was a common complication after skin surgeries; however, it is far away to be classified as a complication related to anaesthesia, and it could happen either under general or local anaesthesia [14].

Regarding the appropriateness and timing of the use of general anaesthesia for children in surgical dermatology, authors and surgeons have settled that general anaesthesia is safe and appropriate for different pediatric dermatologic operations, regardless of the factor of age [15]. Chen and Eichenfield [16] suggested that timely surgical intervention under general anaesthesia will be the best option in children with lesions that are associated with substantial health risks or may result in deformity or functional impairment if left untreated. Also, children who will definitely benefit from early surgical intervention because of the superior cosmetic outcome which will be obtained [17], as well, in children where the timely surgical correction will positively affect their psychosocial status and self-esteem [18]. Deep sedation and general anaesthesia are safe and cost-effective approaches to control pain. Physicians should encourage parents to do such procedures as early as possible [19].

Before the use of general anaesthesia, many concerns are routinely checked up, such as a healthy patient, appropriate choice of surgery, promising outcome, benefits of early surgical intervention in children, and right anaesthesia setting [11]. The skin of children is much more pliable than adults, with good healing properties. These advantages allow the surgeon to remove large lesions with primary closure [14]. The elasticity of the skin in children plays an important role in healing and ease of manipulation than inelastic skin. Inelastic skin in adults sometimes leads the surgeon performing multiple surgeries, while waiting for better wound healing and skin growth [7].

To avoid postoperative complications such as large scaring, spread scar, or wound dehiscence, surgeons should consider an early intervention at a young age. The skin is elastic and redundant with less muscle mass. This allows easily the usage of skin flaps, tissue expanders, or grafts. Lesion at a young age is not under big tension [16, 17].

In children, sedation is more risky than in adults because sedation levels are not as obviously distinct [20]. Sedation also does not provide characteristic airway protection in comparison to general anaesthesia [21]. Repair procedures and large excisions are too painful to be controlled by light sedation. Also, even a small excision with little pain is difficult to be controlled by light sedation because of the fear of uncooperative behaviour of the child [22]. Death of children has occurred because the anesthesiologists failed to save the airway in spite of safe sedation [23].

Children go beyond one sedation level to another with no clear signs, and that increases the likelihood of risk during the sedation when compared to adults. Moreover, it will be difficult to secure the airway during the procedure performed under sedation, [24] as children have compromised airway due to anatomical variation that makes their airway more prone to obstruction or injury. In addition, the relatively large tongue and small oral cavity make the procedure of accurate evaluation difficult. Other factors make the process of airway protection in children impossible, such as prominent laryngeal and pharyngeal structures, short trachea, and neck flexion due to projecting occipital bulge [25].

Sedation may be valuable in case of minor procedures with short duration, provided that there is an adequate number of well-trained personnel and equipment to ensure the safety and proper management of the airway [22]. On the other hand, procedures concerned with excision and repair should be performed under general anaesthesia. Generally, the usage of general anaesthesia in pediatric surgeries is favourable and safer than sedation [26].

Trained anesthesiologists ensure the proper position of the endotracheal tube by proper positioning of the patient's head, to avoid displacement of the tube in to the right bronchus or dislodgement. Trained staff, beside their experience of at least 250 pediatric performed cases per year, have a lower incidence of anaesthesia-related complications compared with children treated by nontrained anesthesiologists [17]. Associated comorbidities, prolonged procedures, and lack of trained staff increased the anaesthesia-related complications by 300% [18]. The usage of the fast-acting anesthetic agent by inhalation was associated significantly with low risk of general anaesthesia.

General anaesthesia and tumescent local anaesthesia in dermatosurgery in infants is a suitable outpatient treatment option for small lesions without any major risks or side‐effects and the benefit of prolonged postoperative analgesia [27]. Because tumescent local anaesthesia (TLA) utilizes a mixture of highly diluted local anesthetics in a carrier solution and is used for skin biopsies, excision of skin tumors, and vein surgery [28], it is completely safe [29].

### 4.1. Limitations of the Study

The study was a retrospective cohort and was conducted in a single tertiary center, it did not involve multicenter approach, and also the small sample size used may contribute to some bias. However, it was an interesting overview of complications and indications for general anaesthesia in dermatosurgery in children. As altogether few papers have been published on this specific patient population, a data analysis seems to be important, even if the presented work has only been analyzed retrospectively.

## 5. Conclusion

In case of proper deploying of staff experience, appropriateness of choice of surgical procedure, patient selection, and modern technology as nondependent confounding variables, dermatologist and pediatricians can safely perform necessary procedures under general anaesthesia with the supervision of pediatric-trained anesthesiologists while considering other safety and risk precautions and the pediatric age group.

## Figures and Tables

**Table 1:** Interpretation of the strength of agreement according to *K* value.

Value of *K*	Strength of agreement
<0.20	Poor
0.21–0.30	Fair
0.31–0.40	Moderate
0.41–0.60	Good
0.61–0.80	Very good
0.81–1.00	Excellent

**Table 2:** Patient characteristics.

Characteristics	Finding
Sex, no. (%)	
Male	111 (52.6)
Female	100 (47.3)
Age	
Mean	4.7 years
Range	4 months–17 years
Total number of procedures	211
Total number of diagnoses	19

**Table 3:** Distribution of patients according to diagnoses.

Diagnosis	No. of patients (%)
Congenital melanocytic nevus	49 (23)
Nevus sebaceous	45 (21)
Port wine stain	41 (19)
Infantile hemangioma	16 (8)
Dermoid cyst	14 (7)
Pilomatricoma	8 (4)
Juvenile aponeurotic fibroma	5 (2.3)
Halo scalp ring	5 (2.3)
Cyst	5 (2.3)
Epidermal nevus	4 (1.9)
Vascular malformation	3 (1.4)
Dermatofibroma	2 (0.9)
Atypical nevus	2 (0.9)
Myofibromatosis	2 (0.9)
Verruca vulgaris or condyloma	2 (0.9)
Spitz nevus	2 (0.9)
Nevus	2 (0.9)
Pyogenic granuloma	2 (0.9)
Foreign body	2 (0.9)
Total	211

**Table 4:** List of complications related to general anesthesia.

No.	Complications	No. of patients (%)	*P* value
1	Sore throat, nausea, and vomiting	4 (33)	0.04
2	Mild-to-moderate frontal headache	3 (25)	0.02
3	Minor trauma to the teeth and lips	2 (17)	0.002
4	“Bruising” from IV injection	1 (8.3)	0.01
5	Painful neck muscles	1 (8.3)	0.008
6	Recall of unpleasant dreams, and return to consciousness	1 (8.3)	0.05
	Total	12/211 (5.6)	0.029

**Table 5:** Prespecified adopted criteria against which the complications were evaluated.

Criteria	No. of patients
Lasted more than 3 hours either by observation or medication	0
Caused annoyance, discomfort, and anxiety to the patient	0
Affected the resumption of normal activities on returning home	0
Resolved by analgesia or medication	2
Patient complained from the complication	2
Patient complained only when asked and interviewed	10
Settled with observation	10
Constitute no threat to the long-term health and well-being of the patient	12

## Data Availability

The quantitative and qualitative data used to support the findings of this study are available from the corresponding author upon request.
